# Perspectives on the Doctor of Public Health (DrPH) education among students and alumni in the United States: a cross-sectional national online survey

**DOI:** 10.1186/s12889-023-16402-3

**Published:** 2023-08-16

**Authors:** Chulwoo Park, Cindy Delgado, Ans Irfan

**Affiliations:** 1 Department of Public Health and Recreation, San José State University, San Jose, CA USA; 2 Claremont Graduate University, Claremont, CA USA; 3 Department of Environmental and Occupational Health, The George Washington University, Washington D.C, USA

**Keywords:** Association of Schools and Programs of Public Health (ASPPH), Council on Education for Public Health (CEPH), Doctor of Public Health (DrPH), Survey data analysis, Cross-sectional study, DrPH students, DrPH alumni

## Abstract

**Background:**

This study explored the current and desired identity of the DrPH degree, focusing on whether the competencies set by the Council on Education for Public Health (CEPH) adequately prepare DrPH graduates for effective public health practice. Additionally, the study investigated the necessity of standardization in DrPH training, referring to a consensus-driven approach that equips future public health practitioners with practical skillsets applicable in real-world scenarios.

**Methods:**

A national cross-sectional online survey titled “National DrPH leaders & practitioners needs assessment” was conducted from November 2020 to February 2021. The survey was based on a self-report by DrPH students and DrPH professionals, consisting of the following two main components: (1) how their DrPH training aligns with CEPH competencies and (2) how they perceive the identity of the DrPH degree. Convenience sampling was used to collect the data, which may have limited representation for all DrPH institutions in the United States.

**Results:**

A total of 222 participants (140 current DrPH students and 82 alumni) completed the survey. The mean of the 10-point Likert scale for the degree to which the DrPH training aligns with 26 CEPH competencies (1: not at all – 10: absolutely) ranged from 6.3 (SD: 2.78) to 7.96 (SD: 2.16). The majority of participants (191/222, 86.04%) were satisfied with the knowledge and skills reflected in their training based on the CEPH competencies. However, more than half of the participants (117/222, 52.70%) sought additional professional development/training outside their institutions. DrPH leaders and practitioners faced barriers where the value of their work might not be fully recognized and endorsed. Participants indicated that the DrPH education should be further distinguished from the PhD education.

**Conclusions:**

The DrPH degree holds significant value within the academic sphere of public health practice in the United States. However, its distinction from PhD programs poses a challenge for employers and organizations in the field, requiring attention from higher education programs. By solidifying the DrPH’s identity, graduates can effectively address diverse public health issues and contribute to creating a safe and healthy environment, including addressing the challenges posed by the COVID-19 pandemic.

## Introduction

### Origin and identity of the DrPH program

The DrPH program in the United States was first established in 1909 at Harvard School of Medicine as the terminal degree for the field of public health [1], designed to address gaps between research and practice with a foundational set of skills in public health, research methods, and communication. The DrPH training aims to create transformative leaders with evidence-based public health practice and research skills [2, 3]. This academic program is intended for professionals who have extensive experience [4, 5] in supervisory roles and are seeking to advance their skills at the executive level using research, policy, evaluation, theory, and quantitative and qualitative analysis to solve complex public health problems. Program lengths can vary from three to seven years with required components for graduation, such as practicum, teaching experience, oral and/or written exams, a professional portfolio, experiential learning, and leadership. However, a consistent challenge that DrPH programs face is how to distinguish their focus and design from other graduate degrees and programs in public health, such as a Master of Public Health (MPH) and Doctor of Philosophy (PhD).

An MPH historically had five public health core knowledge areas: epidemiology, biostatistics, social and behavioral sciences, health services administration, and environmental health. Courses at this master’s level were designed to provide an overview of these core areas but in “less-than-optimal depth and rigor” [6] to assist individuals in initiating a career in public health. These core areas have informed the MPH foundational competencies: (1) evidence-based approaches to public health, (2) public health & health care systems, (3) planning & management to promote health, (4) policy in public health, (5) leadership, (6) communication, (7) interprofessional practice, and (8) systems thinking [7]. DrPH programs, while including those MPH’s five-public core knowledge areas and foundational competencies, also have additional competencies in the following areas: (1) data and analysis, (2) leadership, management, and governance, (3) policy and programs, and (4) education and workforce development [7]. These additional competencies equip students with a breadth of skills to lead in diverse settings. For example, courses at the DrPH level have a leadership focus [5, 8, 9] and are designed to dive deeply and rigorously into solving complex public health problems.

A DrPH degree is not to be confused with a PhD degree in public health, a research-focused degree intended to support individuals in research and teaching roles in academia. Courses for PhD programs tend to have a greater emphasis on developing rigorous research skills and dissemination of research. The DrPH has a greater emphasis on leadership experience, as many of the DrPH programs require applicants to have a few years of prior working experience in managerial or leadership roles. Additionally, the degree requires a DrPH integrative learning experience; DrPH candidates need to gain and create an advanced level of field-based products, which makes the DrPH degree unique from a PhD degree. Furthermore, DrPH programs pursue a transdisciplinary approach, developing a shared conceptual framework by integrating and extending discipline-based concepts, theories, and methods [9], while most PhD programs focus on specific areas of study.

### Ongoing efforts to standardize the DrPH training

A constant challenge in the last few decades has centered on identifying ways to provide focused competency training in the program to address workforce gaps among DrPH leaders [10, 11]. After World War II, the emphasis on preventive and social medicine, which had originated in England, started to be applied to schools of public health in the United States, with the aim of bridging the gap between clinical and social medicine [12–14]. Consequently, the American Public Health Association (APHA) accredited graduate professional education in public health between 1945 and 1973 [12]. This accreditation prompted the establishment of the Association of Schools of Public Health (ASPH) in 1953, aiming to enhance education, research, and service in public health [15]. Additionally, the Council on Education for Public Health (CEPH) was formed in 1974 to address the discrepancies in training [12]. ASPH released the agreed-upon national Core Competency Model for the DrPH degree in 2009 to ensure that DrPH programs meet specific educational standards and prepare graduates for developing leadership management and research skills in public health [16]. After that, DrPH Expert Panel was created in 2014 to further address the future directions of the DrPH degree [17]. This Expert Panel analyzed changes in the DrPH based on the DrPH Competency Model. There were three key takeaways from this panel: (1) addressing the need for DrPH programs and graduates to influence decision-making at community, institutional, and governmental levels, (2) incorporating training in communication strategies, evidence-based policy development, and analysis, (3) including ethical and human rights frameworks and foundations in the curriculum [2]. In 2013, ASPH was renamed to the Association of Schools and Programs of Public Health (ASPPH) to reflect a broader membership and design a common strategic framework for public health education [18]. The CEPH’s accreditation criteria for a DrPH program, including DrPH core competency model, has been continuously developed and were subsequently amended in 2016 [19] in 2021 [20]. The inclusion of DrPH competencies as an accreditation requirement signifies the importance of a standardized set of skills and knowledge for individuals pursuing this advanced degree in public health.

The DrPH degree was found to have diverse training formats (in-person versus online program), curriculum demands (practicum, teaching experience, differing dissertation formats), and program length (ranging from 3 to 7 years) [21], with the allowed time for completion of up to 9 or even 12 years. These differences, historically, have continued to create educational training and practice-based challenges in clearly defining the role and purpose of the DrPH degree in public health. Recently, as a result of varying program requirements and needs, a total of 10 DrPH offerings between 2017 and 2019 were discontinued. Reasons for suspension included changing a degree name from DrPH to PhD without any major curricular changes (n = 3), low student interest and enrollment (n = 3), low student interest, as well as faculty and curriculum that aligned more with the PhD (n = 1), and planning to reinstate or revise DrPH (n = 3) [22]. The lack of standardization and understanding of the degree in educational institutions creates discrepancies in training transdisciplinary leaders for public health practice. Consequently, the absence of clear comprehension of the disparate DrPH workforce needs and skills can lead to additional challenges when trying to adequately prepare leaders in public health.

For instance, the COVID-19 pandemic has demonstrated the need for diverse leaders to effectively assist vulnerable communities. To adequately prepare future leaders in public health, a greater emphasis on a transdisciplinary standardized core of leadership in public health practice is needed. However, the demand for practice-based leaders in public health has not been fully met due to a low number of DrPH graduates; the number of annual completion of a DrPH degree has remained low (ranging from 126 to 279 per year) compared to the combined number of all other doctorates in public health (i.e., PhD, ScD, and Joint/Dual) over the last 10 years (ranging from 735 to 1,435) [12, 23]. During the COVID-19 pandemic, training and recruiting the public health workforce has become especially essential to the public health system and infrastructure, and DrPH graduates can help compensate for staffing shortages [24].

Efforts to improve DrPH training have persisted in equipping public health leaders with advancements in leadership skills and evidenced-based research translation. Additionally, it is essential to note that the focus of this study is on DrPH programs in the United States, given the direct applicability and jurisdiction of CEPH. However, the challenge of standardizing public health practice and leadership is global, with similar discourse [25] taking place in South Africa [26–28], India [29], China [30], Korea [31], Southeast Asia [32], Ethiopia [33], and Pan Africa [25]. Many identity-defining efforts have been made by other accreditation agencies, such as the United Kingdom Public Health Register [34], the Board Certified in Public Health (CPH) [35], the National Consortium for Public Health Workforce Development [36], and the Council on Linkages Between Academia and Public Health Practice [37]. Although there have been ongoing efforts to standardize the DrPH training both domestically and globally, a consensus on the ideal direction of DrPH education has not yet been reached. This article focused on the DrPH education and its CEPH competencies in the United States.

### Examples of DrPH program developments with different approaches

Park and Shimada (2022) organized the list of DrPH programs in the United States by the institution name, college/school name, program classification (i.e., major, specialty, discipline, or concentration), mode of instruction, and enrollment type [38]. The findings from various DrPH programs illustrate different approaches to program design and structure. These examples include hybrid learning models, online part-time enrollment, school-wide professional programs, and DrPH-tailored dissertations. For instance, the University of South Florida (USF) utilizes a hybrid program that emphasizes leadership skills and evidence-based practices, offering a mix of online coursework and on-campus summer institutes [39]. The University of North Carolina (UNC) offers an online part-time program with a focus on leadership development, practice-based research, and a dissertation that incorporates a plan for change [40, 41]. Harvard T.H. Chan School of Public Health (HSPH) redesigned its program to be a school-wide professional program, emphasizing competency domains and interdisciplinary learning [8]. The University of Illinois at Chicago School of Public Health (UIC-SPH) distinguishes its DrPH program through a portfolio assessment, instead of traditional qualifying exams, and focuses on a practice-based dissertation using mixed methods research. These varied program designs cater to mid to senior-level professionals seeking to bridge the gap between research and practice, addressing organizational-level issues in public health [5].

### DrPH program developments at the global level

Efforts aimed at addressing the complexity of public health education have centered around shifting away from a narrow focus solely on disease risk and embracing broader and integrative learning approaches. In order to achieve this, Neuhauser et al. (2007) emphasized the importance of employing interdisciplinary, transdisciplinary, and translational approaches to public health education, enabling students to effectively navigate various systems [9]. However, to facilitate such training, they highlighted the necessity of having transdisciplinary scientists who have undergone similar education and training [9]. The development of transdisciplinary leaders necessitates innovative research approaches, educational designs, and the incorporation of collaborative and international partnerships to effectively tackle intricate problems.

Globally, endeavors have been made to incorporate the viewpoints of public health leaders in the development of education and healthcare systems. For instance, a qualitative study conducted in South Africa’s health system revealed that public health stakeholders perceived the public health profession to have a low standing due to an unclear identity [26, 27]. In order to enhance the field, Zweigenthal et al. (2018 & 2019) advocated for a curriculum redesign to improve recruitment efforts [26, 27]. In China, the significance of DrPH training has been acknowledged as a means to address complex problems and challenges [30]. Particularly, there is a need to promote the degree, as DrPH graduates possess expertise in “complex problem solving, organizational leadership, and prevention,” thereby facilitating an international approach to effectively respond to outbreaks. In Africa, a mixed-methods study involving perspectives from public health professionals and future DrPH degree holders emphasized the necessity for establishing a Pan-African DrPH program. Among the identified competencies for the Pan-African DrPH, individuals have expressed interest in courses that focus on leadership, change and systems management, adaptation, and organizational skills, fostering the development of a shared vision [25]. In terms of the design of the DrPH program, public health perspectives have emphasized the importance of a program that prioritizes strategic leadership and incorporates a practice-based learning model, facilitating the integration of theory and practice. These examples not only highlight the global demand for and interest in the DrPH degree but also underscore the necessity for standardization to enhance comprehension of the skills that public health leaders contribute to the field.

### Current and future DrPH training perspectives

The most recent assessment of the DrPH degree in the United States was conducted by Park et al. (2021) [12]. The distribution of DrPH graduate outcomes by employment sector during 2016–2018 was mainly academic institutions (26%), government (17%), for-profit organizations (10%), healthcare organizations (8%), and non-profit organizations (6%) [12]. Aligning with this distribution, potential roles that DrPH holders can have include but not limited to academic faculty, research scientist, and administrator (academic institution); public health director/commissioner, health policy analyst, and epidemiologist (government); health program manager, public health consultant, and healthcare administrator (for-profit organization or non-profit organization); and healthcare administrator (healthcare organization). Wide variances in DrPH programs in the United States have been an ongoing challenge [12], showing a situation similar to past research conducted over a decade ago [3, 16]. To overcome scattered interpretations of the DrPH curriculum across the nation, Park et al. (2021) called for a clear DrPH identity and standardized training to improve workforce development among DrPH leaders [12]; this research used DrPH Directors’ experiences to assess the status and development of the DrPH degree. However, it is important to note that as of February 2020, only 30% (15/50) of the DrPH Directors from the 28 CEPH-accredited DrPH programs earned a DrPH degree for their doctoral study [3, 42]. The lack of inclusion of DrPH directors who experienced the DrPH education for their own terminal degree could give rise to confusion in establishing the identity of a DrPH degree when they develop and design DrPH curriculums and programs. Even though DrPH students and alumni are actual recipients of the DrPH education, those previous studies did not include them to assess DrPH programs [12, 42]. In fact, those articles were limited to the professional opinions of academic heads of their respective programs (i.e., DrPH directors) [12, 42]. To expand on research findings, we conducted a national cross-sectional online survey to gather perceptions from DrPH students and alumni in the United States. Thus, this study aimed to analyze the current and desired identity of the DrPH degree by investigating whether CEPH competencies have prepared DrPH graduates to gain both theoretical and practical skillsets that can be readily applied in real-world scenarios. Furthermore, we sought to delineate the concept of standardization within the context of DrPH training, which entails a nationwide consensus-driven approach to curriculum development. The aim is to equip aspiring public health practitioners. It is worth noting that this standardization of the DrPH degree differs from the primary focus of a PhD degree, which centers on theoretical research methodologies.

## Methods

### Study design

A national cross-sectional online survey titled “National DrPH leaders & practitioners needs assessment” was conducted among DrPH students and alumni from November 2020 to February 2021 to assess and explore their perceptions related to (1) self-identified current level of DrPH foundational competencies set by CEPH and (2) DrPH identity. The survey had two goals. First, we sought to identify whether a movement was needed to further establish the DrPH identity. The second goal was to identify if standardization of the DrPH training was desired. The survey was collected through Google Forms hosted on a secure server. The authors consisted of two DrPH alumni (CP: started in 2016, graduated in 2020; AI: started in 2018, graduated in 2021) and one DrPH student (CD: started in 2017) at the time of research, all of whom had expertise in DrPH education and development of the survey instrument. All of them started their DrPH education after CEPH DrPH competencies were introduced.

### Setting and participants

We used a nonprobability sampling technique, the snowball convenience sampling approach as a network-based convenient sample [43], to recruit participants who self-identified as public health practitioners and leaders, without using a strict definition of public health leaders and practitioners. We contacted a total of 36 DrPH directors from CEPH-accredited DrPH programs in the United States to request the dissemination of recruitment materials through their social media (e.g., Twitter and LinkedIn), membership lists, and an email listserv. Subsequently, we reached out to national public health organizations, such as the Student Assembly from APHA and the DrPH Coalition. Thus, our sample was dependent on the authors’ professional contacts and resources. The eligibility criteria for participation were restricted to self-identified current DrPH students or alumni in the United States, without a restriction on the year of graduation. Therefore, the main purpose of this study was to focus on those individuals who indeed have/had experienced with the DrPH curriculum as a student in the United States to better understand potential implications for the DrPH identity and future public health workforce development in the United States.

### Data collection and measurement

The total number of participants who completed the survey was 222 (current DrPH students: 140; alumni: 82). Data collection started on November 23, 2020, and stopped on February 15, 2021. According to the Accreditation Criteria amended in October 2016 [7], CEPH introduced 20 foundational competencies through the following domains: (a) Data & Analysis (3 competencies), (b) Leadership, Management & Governance (10 competencies), (c) Policy & Programs (4 competencies), and (d) Education & Workforce Development (3 competencies). Participants were asked how well their DrPH training prepared them to achieve each of the CEPH competencies. We defined “DrPH Training” as the training that students or alumni are/have/had received in their DrPH program. The 1–10 rating scale (1: not at all, 10: absolutely) was used to measure the level of alignment between DrPH training and CEPH competencies. A total of 26 questions (6 questions from Data & Analysis, 13 questions from Leadership, Management, & Governance, 4 questions from Policy & Program, and 3 questions from Education & Workforce Development) were extracted by adding a prompt, “Has your DrPH training prepared you to …?” to each of the original CEPH competency sentences (e.g., “Has your DrPH training prepared you to design a system-level intervention to address a public health issue?”). If a CEPH competency sentence contains more than one component, we further dissected that competency sentence into more than one survey question to ask each component. For example, one of the CEPH competencies from the domain of Leadership, Management & Governance, “Assess one’s own strengths and weaknesses in leadership capacities, including cultural proficiency,” was divided into two survey questions to separately ask about “leadership capacities” and “cultural proficiency.”

In addition, we measured the participants’ level of establishment of DrPH identity via four domains: (1) perceptions of competency training, (2) distinction from PhD, (3) recognition, and (4) standardization. Two types of questions were used: Likert scale questions asked the range of 1–10, and binomial questions asked Yes or No.

### Statistical methods

To analyze survey data, we used Stata/MP 14.2 (StataCorp, LLC, College Station, TX). Once the survey period was finished, the collected data from Google Forms were exported as Microsoft Excel (.xlsx) and then imported into Stata. Demographic statistics were conducted to measure the characteristics of the survey participants. To measure the level of current alignment between DrPH training and CEPH competencies, we used means and standard deviations (SD) for a 1–10 rating scale and then compatibility intervals (CI) for mean differences. Cronbach’s alpha was used to measure the internal consistency of each domain as well as across all domains from CEPH competencies. In addition, the Pearson correlation matrix was used to measure a linear correlation between two sets of those domains. For the level of current or desired establishment on DrPH identity, both binary and 1–10 rating scale questions were used. Two-tailed paired t-tests were conducted when comparing two questions that provided significant mean differences.

### Human subject protections

The research study was approved by the San José University Institutional Review Board (IRB Protocol Tracking Number: 20,270) as part of a larger mixed-methods study focusing on DrPH students’ and alumni’s needs assessment and future directions. Respondents read and consented to an electronic consent form before proceeding with completing the survey.

## Results

### Demographic information

Table 1 shows the demographic information of the participants. More than half of the participants were current DrPH students (140/222, 63.06%). There were various ways that individuals are funded for their DrPH education: federal student loans (32.43%), scholarship/assistantship/fellowship (24.32%), self-financed (22.52%), employment benefits (3.6%), private loans (1.8%), veteran education benefits (1.35%), and combination any of funds (13.96%). In addition, participants had the option to select or self-identify their professional background, primary goal, and sector that they plan to work in from the list, and they were able to check boxes of non-mutually exclusive categories. More than half of them had a background in public health practice (137/222, 61.71%), aligning with their primary goal after DrPH graduation: public health practice/leadership (177/222, 79.73%). However, over a third (79/222, 35.59%) of the participants also identified ‘public health research’ as a part of their professional background. The top seven sectors that participants planned to work in after graduation were (1) federal government/military, (2) non-profit, (3) NGOs, (4) academia/higher education, (5) state health departments, (6) consulting, and (7) local health departments.

**Table 1:** Demographic information of participants (Sample size, N = 222)

Characteristic	N = 222 No. (%)	Characteristic	N = 222 No. (%)
**Status**		**Where are you located**	
Current DrPH student	140 (63.06)	West	57 (25.68)
DrPH alumni	82 (36.94)	Midwest	19 (8.56)
**Age**		South	64 (28.83)
21–25	4 (1.80)	Northeast	66 (29.73)
26–30	27 (12.16)	Outside the Continental U.S.	5 (2.25)
31–35	61 (27.48)	Non-U.S. (International)	7 (3.15)
36–40	38 (17.12)	Prefer not to say	4 (1.80)
41–45	33 (14.86)	**Employment status**	
46–50	29 (13.06)	Full-time	170 (76.58)
51–55	11 (4.95)	Part-time	41 (18.47)
56–60	14 (6.31)	Do not work	9 (4.05)
61–65	4 (1.80)	Prefer not to answer	2 (0.90)
Older than 65	1 (0.45)	**Years of full-time-equivalent work experience**	
**Gender**		1–2 years or less	11 (4.95)
Man	53 (23.87)	3–5 years	25 (11.26)
Woman	166 (74.77)	5–10 years	60 (27.03)
Transman	1 (0.45)	More than 10 years	126 (56.76)
Nonbinary	1 (0.45)	**Household income**	
Prefer not to answer	1 (0.45)	Less than $10,000	3 (1.35)
**Race**		$10,001 - $25,000	10 (4.50)
White	106 (47.75)	$25,001 - $50,000	15 (6.76)
Black	65 (29.28)	$50,001 - $75,000	25 (11.26)
Asian or Pacific Islander	26 (11.71)	$75,001 - $100,000	30 (13.51)
American Indian/Alaskan Native	5 (2.25)	More than $100,000	117 (52.70)
Prefer not to answer	6 (2.70)	Prefer not to answer	22 (9.91)
Other	14 (6.31)	**Payment for DrPH education (multiple responses)**	
**Hispanic**		Federal student loans	72 (32.43)
Yes	40 (18.02)	Scholarship, assistantship, fellowship	54 (24.32)
No	178 (80.18)	Self-financed	50 (22.52)
Prefer not to answer	4 (1.80)	Employment benefits	8 (3.60)
**Marriage**		Private loans	4 (1.80)
Yes	129 (58.11)	Veteran education benefits	3 (1.35)
No	92 (41.44)	Combination of above	31 (13.96)
Prefer not to say	1 (0.45)	**Professional background (multiple responses, top 20)**	
**Raising any children**		Public health practice	137 (61.71)
Yes	101 (45.50)	Public health research	79 (35.59)
No	118 (53.15)	Prevention and community health	53 (23.87)
Prefer not to answer	3 (1.35)	Health policy	41 (18.47)
**What is/was your primary goal after DrPH graduation?**		Global/International health	38 (17.12)
Public health practice/leadership	177 (79.73)	Epidemiology	33 (14.86)
Research & academia	45 (20.27)	Healthcare administration	29 (13.06)
**What sector currently/did you plan to work after graduation?**		Health systems research	16 (7.21)
Federal government/military	51 (22.97)	Environmental health	15 (6.76)
Non-profit	40 (18.02)	Mental health	15 (6.76)
NGOs	26 (11.71)	Entrepreneurship	12 (5.41)
Academia/higher education	24 (10.81)	Biology	11 (4.95)
State health departments	21 (9.46)	Business	11 (4.95)
Consulting	21 (9.46)	Emergency preparedness	11 (4.95)
Local health departments	12 (5.41)	Biostatistics	10 (4.50)
Biopharmaceuticals	6 (2.70)	Social work	10 (4.50)
Healthcare	3 (1.35)	Other clinician (PA/Nursing/Dentist/Oral health)	9 (4.05)
International health organizations	3 (1.35)	Clinical Medicine (MD)	8 (3.60)
Philanthropy	2 (0.90)	Humanitarian aid	8 (3.60)
Clinical	1 (0.45)	Occupational health	8 (3.60)
Community-based organizations	1 (0.45)	*Note: Column totals greater than 100% due to multiple responses for this item.	
To be determined	11 (4.95)		

### DrPH training’s alignment with CEPH competencies

Table 2 displays the level of current alignment between DrPH training and CEPH competencies measured by participants. The mean scores (possible range: minimum 1, maximum 10) were calculated for each of the 26 questions assessing DrPH training aligned with the four CEPH competency domains. The mean scores for each category were all greater than 6: (1) Data & Analysis (ranged from 7.3 to 7.96), (2) Leadership, Management & Governance (ranged from 6.3 to 7.95), (3) Policy & Programs (ranged from 7.41 to 7.72), and (4) Education & Workforce Development (ranged from 6.98 to 7.85). Cronbach’s alpha was greater than 0.9 across all four domains, indicating that the questions in each domain were highly correlated. Table 3 shows the interrelationships among four domains from 20 CEPH competencies. All of the correlations were significant with the anticipated direction (*P* < 0.001, the range of Pearson’s *r*: 0.7913–0.9039).

**Table 2:** The level of current alignment between DrPH training and CEPH competencies

Domain	Question: “Has your DrPH training prepared you to … ?” (1–10 rating scale; 1: not at all – 10: absolutely)	Mean (SD)	Cronbach’s alpha
Data & Analysis (6 questions)	… design a qualitative research project to address a public health issue?	7.96 (2.16)	0.9003
	… design a quantitative research project to address a public health issue?	7.72 (1.96)	
	… design a mixed-methods research project to address a public health issue?	7.55 (2.19)	
	… design a health policy analysis project to address a public health issue?	7.4 (2.45)	
	… design a program evaluation project to address a public health issue?	7.78 (2.06)	
	… explain the use and limitations of surveillance systems and national surveys in assessing, monitoring, and evaluating policies and programs and to address a population’s health?	7.3 (2.29)	
Leadership, Management & Governance (13 questions)	… propose strategies for health improvement and elimination of health inequities by organizing stakeholders, including researchers, practitioners, community leaders, and other partners?	7.73 (2.31)	0.9693
	… communicate public health science to diverse stakeholders, including individuals at all levels of health literacy, for purposes of influencing behavior and policies?	7.62 (2.29)	
	… integrate knowledge, approaches, methods, values, and potential contributions from multiple professions and systems in addressing public health problems?	7.95 (2.07)	
	… create a strategic plan?	7.32 (2.56)	
	… to facilitate shared decision making through negotiation and consensus-building methods?	7.08 (2.55)	
	… create organizational change management strategies?	7.13 (2.57)	
	… to propose strategies to promote inclusion and equity within public health programs, policies, and systems?	7.22 (2.58)	
	… assess your own strengths and weaknesses in leadership capacities?	7.89 (2.44)	
	… assess your own strengths and weaknesses in cultural and structural proficiency?	7.28 (2.64)	
	… propose human resources to achieve a strategic goal?	6.68 (2.72)	
	… propose fiscal resources (e.g., multimillion budgets for organizations) to achieve a strategic goal?	6.3 (2.78)	
	… find and propose multiple resources to achieve a strategic goal?	7.1 (2.57)	
	… cultivate new resources and revenue streams to achieve a strategic goal?	6.4 (2.8)	
Policy & Programs (4 questions)	… design a system-level intervention to address a public health issue?	7.59 (2.29)	0.9208
	… integrate the knowledge of cultural values and practices in the design of public health policies and programs?	7.41 (2.39)	
	… integrate scientific information, legal and regulatory approaches, ethical frameworks, and varied stakeholder interests in policy development and analysis?	7.55 (2.35)	
	… propose interprofessional team approaches to improving public health?	7.72 (2.41)	
Education & Workforce Development (3 questions)	… assess an audience’s (community partners, professional audience, students) knowledge and learning needs?	7.66 (2.35)	0.9086
	… deliver training or educational experiences that promote learning in academic, organizational, or community settings?	7.85 (2.22)	
	… use the best practice modalities in pedagogical practices in a wide range of settings?	6.98 (2.72)	
Single factor Cronbach’s$\alpha$			0.9798

**Table 3:** Pearson correlation matrix of four domains from 20 CEPH competencies

Domains	1. Data & Analysis	2. Leadership, Management & Governance	3. Policy & Programs	4. Education & Workforce Development
1. Data & Analysis	—			
2. Leadership, Management & Governance	0.7913*	—		
3. Policy & Programs	0.8252*	0.9039*	—	
4. Education & Workforce Development	0.8077*	0.8774*	0.8946*	—

* *P* < 0.001

### Establishment of DrPH identity

Table 4 demonstrates the current and desired level of establishment of DrPH identity measured by participants, consisting of three main topic areas: (1) perceptions of competency training, (2) distinction from PhD, and (3) DrPH recognition and standardization.

**Table 4:** The level of current or desired establishment on DrPH identity

Domain	Question	Yes/No (%)	Mean (SD)
Perceptions of competency training (3 questions)	Do you think CEPH competencies adequately reflect the skills needed for a trained public health workforce?	Yes: 191 (86.04) No: 31 (13.96)	
	How well do you think your current DrPH program meets the CEPH competencies? (Scale 1: not at all – Scale 10: completely)		7.75 (2.08)
	Have you pursued professional development/training opportunities outside of your DrPH program to meet any of the CEPH competencies?	Yes: 117 (52.70) No: 105 (47.30)	
Distinction from PhD (2 questions)	In your view, nationally, how distinct IS the DrPH training compared with a PhD? (Scale 1: same as PhD – Scale 10: totally distinct)		5.68 (2.61)
	In your view, how distinct SHOULD the DrPH training be compared with a PhD? (Scale 1: same as PhD – Scale 10: totally distinct)		6.59 (2.8)
Recognition (4 questions)	Does your professional network perceive the DrPH as a prestigious degree? (Scale 1: not at all – Scale 10: very prestigious)		6.91 (2.26)
	Do you think the DrPH is a well-recognized degree to the public? (Scale 1: not at all – Scale 10: well-recognized)		5.72 (2.29)
	In your assessment, how knowledgeable are employers about DrPH training? (Scale 1: not at all – Scale 10: very knowledgeable)		4.28 (2.16)
	In your view, how much do public health employers/organizations value the distinct DrPH training in leadership and management? (Scale 1: not at all – Scale 10: super valued)		5.78 (2.25)
Standardization (4 questions)	Do you think DrPH should provide standardized training?	Yes: 173 (77.93) No: 49 (22.07)	
	Do you think the DrPH should have a standard core curriculum across the nation?	Yes: 177 (79.73) No: 45 (20.27)	
	Do you think the DrPH should have standard training across the nation like an MD or JD?	Yes: 162 (72.97) No: 60 (27.03)	
	Do you think DrPH should have a standard exam like an MD Board Exam?	Yes: 70 (31.53) No: 152 (68.47)	

#### Perceptions of competency training

We conducted a chi-square test of independence test to identify if there is a difference in perception between students and alumni. The results showed no significant difference between current students (87.14%, 122/140) and alumni (84.15%, 69/82), chi-square χ2(1) = 0.39, *P* = 0.53). Overall, most participants (194/222, 86.04%, answered “Yes” to a binary variable) agreed that the CEPH competencies for the DrPH have adequately reflected the skills needed to train the public health workforce. Although DrPH programs were considered to be successfully meeting the CEPH competencies (mean = 7.75, SD = 2.08 on a 1–10 rating scale, 1: not at all, 10: completely), more than half of the respondents (117/222, 52.7%) had pursued additional professional development or training opportunities externally, either during the process of obtaining the DrPH degree or after its completion. Both students (49.29%, 69/140) and alumni (58.54%, 48/82) had looked for those external opportunities to build up additional abilities not obtained from their DrPH education (chi-square χ2(1) = 1.78, *P* = 0.18).

#### Distinction from PhD

We asked two questions about the distinction between DrPH and PhD, using a rating scale from 1 to 10 (1: same as PhD, 10: totally distinct). The first question, “In your view, how distinct *is* the DrPH training compared with a PhD?” had a mean score of 5.68 (SD = 2.61). The second question, “How distinct *should* the DrPH training be *compared with* a PhD?” had a mean score of 6.59 (SD = 2.8). The result of the two-tailed paired *t*-test had a mean difference of -0.92 (95% CI: -1.32, -0.52), P < 0.001, indicating that participants regarded the DrPH to be similar to the PhD degree and wanted to see their DrPH training distinguished from a PhD.

#### DrPH recognition and standardization

There was a large gap between the level of recognition that a DrPH degree holds among participants’ professional networks versus among external public health stakeholders (the public, employers, and organizations). The mean for the question, “Does your *professional network* perceive the DrPH as a prestigious degree?” (1: not at all, 10: very prestigious) was 6.91 (SD = 2.26). However, the mean scores were significantly lower when participants were asked the following three questions: (1) 5.72 (SD = 2.29) for “Do you think the DrPH is a well-recognized degree *to the public*?”; (2) 4.28 (SD = 2.16) for “How knowledgeable are *employers* about DrPH training?”; and (3) 5.78 (SD = 2.25) for “how much do *public health employers/organizations* value the distinct DrPH training in leadership and management?” These findings imply that the level of recognition of a DrPH degree among external stakeholders was lower than that of DrPH among participants’ own professional networks. We conducted two-tailed paired t-tests to compare the mean of 6.91 for the perception level of a DrPH degree among participants’ professional network with those three means for the recognition level of a DrPH degree among the public (mean: 5.72), employers (mean: 4.28), and public health employers/organization (mean: 5.78). P-values and CIs from each of the t-test results verified that the mean differences were 1.19 (95% CI: 0.95, 1.43), 2.64 (95% CI: 2.31, 2.96), and 1.13 (95% CI: 0.82, 1.44), respectively. None of them included zeros between the lower and upper bounds of 95% CIs, and they were all statistically significant at α = 0.001.

The findings indicated that a significant majority of the participants, with a proportion of 173 out of 222 (77.93%), were in agreement that DrPH education should encompass standardized training. Similarly, 177 out of 222 participants (79.73%) expressed their support for implementing a standard core curriculum within the program. Furthermore, 162 out of 222 participants (72.97%) believed that DrPH education should strive for standard training across the nation, akin to other professional terminal degrees, such as a Doctor of Medicine (MD) or a Juris Doctor (JD) degree. However, only 70 participants (31.53%) agreed that DrPH should include a standardized examination, such as the Medical Board Exam.

## Discussion

Prior research that included perspectives of academic heads of the programs (i.e., DrPH directors) did not show intentions of standardizing DrPH programs across the nation [42]. Thus, this study raised important perspectives from actual recipients of DrPH education. This study builds on existing core competency DrPH literature [16], Roemer’s vision [6] for a practice-based doctoral degree, and Northbridge and Healton’s question of ‘Who Will Deliver on the Promise of the DrPH Core Competency Model?’ [44]. Nearly a decade later, those challenges of aligning DrPH education with the needs of public health practitioners have remained stubbornly the same [4, 45]. Additional literature on the development of specific DrPH programs presenting case studies, such as the University of California, Berkeley [9], the University of South Florida [39], and Harvard University [8], also highlighted similar challenges of bridging the gap between academia and practice.

This study was one step forward in better understanding the needs of the DrPH alumni and DrPH students to assist standardization efforts and improve the identity of the degree. Results from the survey demonstrate that among those individuals that participated in the study, the majority felt that the CEPH competencies adequately reflected the skills needed to be trained in the public health workforce. However, an interesting finding is that more than half of the participants looked for professional development/training opportunities outside of their DrPH program to meet the CEPH competencies. One possible consideration for this result is the changing landscape of public health and the diverse skills needed to solve complex problems. The need for transdisciplinary skills—such as combining elements from the disciplines of organizational change, implementation sciences, and strategic planning—in executing solutions to complex public health issues could be another reason why students sought professional development opportunities externally. This could be a reflection of the dissonance between the need for practice-based skills/training and the theory-oriented DrPH education received by the participants. It is important to note that the CEPH requirements for accredited DrPH programs, unlike some other professional degrees such as MD, do not have a standardized practice-based training, residency, or immersion experience requirement. Universities/schools interpret the practice requirement in a variety of different ways, including reflection portfolios, immersions embedded in organizations, and varying numbers of practice-based experiential learning.

In regard to DrPH identity at a national level, participants expressed their training was not significantly distinct from the PhD degree, demonstrating an opportunity for programs to create and develop distinctions between the two degrees. When we asked *how* distinct the two degrees *should* be, participants expressed interest in distinction. This presents an opportunity to consider how the DrPH and PhD degrees can be defined by their unique differences. For example, another professional terminal degree, such as a Doctor of Psychology (PsyD) focuses more on a wide range of practical or clinical aspects of applied skills, while a PhD degree emphasizes research [46]. PsyD in Clinical Psychology at The George Washington University provides clinically-focused training and research methods and requires a yearlong internship [47]. Similarly, the EdD is a program geared towards professionals seeking to advance their leadership skills [48–50]. At the University of Southern California, the EdD program focuses on educational leadership training and utilizes research to provide practical solutions [51]. Their research methods address practical problems that hinder access to educational outcomes and opportunities [49].

Participants felt the DrPH was recognized as a prestigious degree within their public health professional network. However, outside of their network, participants felt that a DrPH degree was not well-recognized. For example, outside the field of public health, there appears to be a persisting stereotype that DrPH programs have less rigorous training than PhD programs. In particular, participants expressed that employers were not knowledgeable about their DrPH training. A marketing strategy thus will be needed to advocate the unique benefits that the DrPH degree brings to the field of public health and beyond.

To meet the needs of DrPH students, DrPH programs should take a leading role in distinction efforts. First, standardization of DrPH’s educational credentials is one possible response to address this identified challenge. Participants strongly agreed that standardization could be the key to creating a clear understanding of the training that DrPH graduates should have. Additionally, participants expressed an interest in standardizing the core curriculum to have a standard national training. Standardizing the curriculum across the nation would not only better support the identity of the DrPH degree but also become a tool for creating a clear distinction from the PhD degree requirements. Second, an educational campaign to inform practitioners and educators about the distinctions of the programs might be another possible approach that was not evaluated extensively. Professional development opportunities offered by ASPPH or APHA could assist not only in educating the public regarding distinctions between PhD and DrPH but also in providing training for DrPH programs to meet some of the critical training needs in key fields and offering lifelong learning in critical current subject areas, such as through Continuing Education Program offered by APHA. Third, DrPH institutions can take community-centered approaches and reflect DrPH students’ and alumni’s needs in class design and curriculum development. Fourth, DrPH institutions can consider sharing lessons learned to distinguish DrPH programs from PhD programs.

The COVID-19 pandemic presents unique challenges that require the expertise of DrPH graduates. It is essential to distinguish COVID-19 from other generalized public health issues, as it has caused significant global disruptions and resulted in substantial loss of life and economic impact [52]. The effects of the pandemic, including high unemployment rates [53, 54], loss of loss of health care insurance [55, 56], delayed care [57, 58], housing [59] and food insecurity [60], and increased domestic violence [61, 62], have had far-reaching consequences. Therefore, future leaders with a DrPH degree must be equipped to tackle multi-level systemic challenges, including the impact of COVID-19. In addition, within a team of public health professionals in an organization or community, individuals with a DrPH can provide the following: leadership and strategic planning, applied research and evaluation, policy development and advocacy, community engagement and collaboration, and program implementation and management.

To address complex public health problems, Welter et al. (2022) discussed the importance of collaborative action, systems change leadership, and health equity and social justice [63]. These components should be considered in curriculum and professional development training for DrPH graduates to better support their workforce needs. Additional components to consider adding to the curriculum include finance, anti-racism and health equity, crisis and emergency management, ethical leadership, and public health law among the practice areas, to prepare public health leaders.

In order to bridge education and public health practice with the DrPH, Ocampo et al. (2020) suggested three solutions [64]. First, training should include systems thinking, business management, communications, and strategy. Second, institutions should provide training in systems thinking, communication, strategy, and political processes to support public health leaders in their roles. Third, opportunities to train with diverse experts (e.g., community leaders, consultants, politicians, environmentalists, and social workers) should be provided to address interdisciplinary problems. To support the training needs of public health professionals, the incorporation of public health topics as well as continuous professional development opportunities are needed. DrPH institutions should provide emerging class topics and include diverse experts to teach those courses comprehensively.

### Strengths and limitations

To date, there has not been a concerted effort to actively involve the DrPH community (students and alumni) in discussions about DrPH’s alignment with CEPH competencies, standardization, identity distinction from PhD, and recognition across employment. This study contributes to the existing DrPH literature in the United States by highlighting the perspectives of the DrPH community based on their academic experiences. This survey provided an overview of the broader spectrum of DrPH stakeholders who were direct beneficiaries of the DrPH education and its core DrPH competencies. We explored whether DrPH alumni and DrPH students want to create the identity and distinction for their degree. In addition, we investigated whether the core competencies for the DrPH education have been applied to their professional development and educational needs.

In this study, several limitations were encountered and should be acknowledged. First, the sample size was limited due to the non-probability convenience sampling method, snowball sampling, which may underrepresent the target population [43, 65], and thus results cannot be generalized to the whole DrPH population [66]. Despite the limitations of snowball sampling, this was still the best method to contact perhaps hard-to-reach alumni and students [67]. Additionally, the convenience of network-based participants could introduce selection bias [43]. However, recruitment expanding to educational institutions and organizations increased our sample size and representativeness. The survey data collected were at the national level, with 222 respondents (140 current DrPH students and 82 graduates). We did not collect the participants’ DrPH institutions to maintain anonymity, but instead collected their current location in the United States. Although most participants were in the Northeast, South, and West of the nation (Table 1), this distribution may not necessarily represent the entire doctoral-granting institutions in the United States offering DrPH programs. To make the results more generalizable and applicable to other countries, future studies could use probability sampling and census data to provide a larger number of perspectives regarding the CEPH competencies and DrPH education. Second, the survey did not include detailed questions on experiential learning (e.g., practicum, residency) which is one of the key components of DrPH programs. Third, this study did not collect data on the year of graduation, which further limits its generalizability. This would limit the applicability of the study’s findings that some of the alumni may have graduated before the CEPH competencies were implemented. Fourth, this study could not distinguish between the field sector in which individuals were already working and the ideal field where they wish to be working in after graduation. To fully understand the training needs of DrPH graduates, identifying this distinction will be essential in creating the unique identity of the DrPH degree while standardizing the curriculum. Lastly, it is important to note that there could be an inherent potential bias in this study, as all the authors are recent recipients of a DrPH degree, and no input was sought from employers or other public health stakeholders who do not hold a DrPH degree. Nevertheless, our study made an initial endeavor to collect the viewpoints of individuals who have obtained the DrPH degree, which would significantly contribute to the future enhancement of the DrPH program by incorporating their feedback.

### Implications and future research

The results from this study reflect the exigency for standardization to improve future training and value for current and future DrPH leaders in the United States. Addressing the perspectives of degree holders and students will be important for creating a clear identity of the DrPH degree. The COVID-19 pandemic has demonstrated the importance of transdisciplinary leaders who create sustainable, equitable, and inclusive solutions to complex public health problems. The core competency model was designed to create “a national discussion” on the competency needs of DrPH students [16]. This model was a historical step in creating standardized training and improving the infrastructure of public health. Northridge and Healton (2012) called on public health programs to “do more than create generalists” and consider the opportunities of the DrPH degree to be more globalized [44].

There is a clear demand for the DrPH degree [68] that emphasizes the need to explore its identity within both community and global contexts in future studies. In response to addressing demands of DrPH education, several potential areas of future research can be pursued. First, further studies can focus on how to establish the DrPH degree as a widely recognized and indispensable practical terminal degree that effectively serves population health needs. For instance, future investigations should delve into identifying any remaining gaps in the implementation of CEPH competencies within DrPH education. Furthermore, it is important to explore the types of additional training that students have pursued to address gaps in their education. Future research will thus need to consider exploring how to build a nationwide direction and understanding of the DrPH degree when designing a standardized training model. Second, future studies can investigate the perceptions about or the need for a DrPH degree from employers and other public health stakeholders who do not possess a DrPH degree. Additionally, these studies can review public health job descriptions to identify the preferred or required degrees for different positions, thereby enhancing our understanding of the gap between the current job requirements and the expertise that DrPH recipients can actually offer.

Lastly, it is important to build upon the DrPH trends during 2017–2019 reported by CEPH [22] and gain an understanding of the overall changes in the DrPH landscape, particularly considering the growing demand for public health workforce during the COVID-19 pandemic. Additionally, given the existence of different types of DrPH programs, such as different format of delivery mode for learning, it is crucial to explore which format of DrPH programs has emerged as the ideal choice, with clear distinctions from PhD programs, for addressing unexpected complex public health issues in the modern era. The format of DrPH programs varies, with part-time online programs catering to a distinct target audience of working professionals, while full-time residential programs require individuals to leave their jobs and relocate. The current demand for DrPH degrees is largely driven by the growth of part-time online programs. Thus, in future studies, it is important to investigate whether this demand is primarily influenced by the feasibility and affordability of pursuing doctoral training in public health while maintaining current employment, rather than being primarily attributed to differences in curriculum between PhD and DrPH programs.

## Conclusions

In the United States, DrPH is the terminal degree for the field of public health. It is well acknowledged and valued within the academic public health practice sphere. However, according to our findings, distinguishing DrPH from PhD programs is a challenge among public health employers and organizations, and it needs to be addressed from a higher education programmatic perspective. Moreover, outside of the participant professional network, DrPH is not widely recognized or distinguished as a unique degree. This is of high concern for effective public health governance and leadership perspectives. The DrPH programs uniquely train public health leaders and practitioners, equipping them with critical public health practice skills. However, from the participants’ perspective, employers do not seem to see its full value. To increase DrPH’s recognition and convey its value to employers, schools and programs of public health, ASPPH, and CEPH should consider focusing on systematic outreach to employers across sectors to create standardization of DrPH training. To progress towards health equity, public health should be led by competent practitioners and leaders. This can be achieved by engaging employers both domestically and globally, emphasizing the value of the DrPH, and placing competent professionals in leadership roles they were trained to fill. By establishing a clear DrPH identity, it is expected that DrPH graduates should be the ones who address urgent, diverse public health issues through a leadership position/role to contribute to creating a safe and healthy public health environment around us, including effective control of the COVID-19 pandemic.

## Data Availability

All data generated or analyzed during this study are included in this published article. The datasets generated and/or analyzed during this study are not publicly available due to the confidentiality of participants’ information but are available from the corresponding author on reasonable request.
